# Multiple functional SNPs in differentially expressed genes modify risk and survival of non-small cell lung cancer in chinese female non-smokers

**DOI:** 10.18632/oncotarget.14836

**Published:** 2017-01-27

**Authors:** Xue Fang, Zhihua Yin, Xuelian Li, Lingzi Xia, Xiaowei Quan, Yuxia Zhao, Baosen Zhou

**Affiliations:** ^1^ Department of Epidemiology, School of Public Health, China Medical University, Shenyang, China; ^2^ Key Laboratory of Cancer Etiology and Prevention, China Medical University, Liaoning Provincial Department of Education, Liaoning, China; ^3^ Department of Radiotherapy, The Fourth Affiliated Hospital of China Medical University, Shenyang, China

**Keywords:** differentially expressed genes, functional single nucleotide polymorphism, non-small cell lung cancer, risk, survival

## Abstract

DNA genotype can affect gene expression, and gene expression can influence the onset and progression of diseases. Here we conducted a comprehensive study, we integrated analysis of gene expression profile and single nucleotide polymorphism (SNP) microarray data in order to scan out the critical genetic changes that participate in the onset and development of non-small cell lung cancer (NSCLC). Gene expression profile datasets were downloaded from the GEO database. Firstly, differentially expressed genes (DEGs) between NSCLC samples and adjacent normal samples were identified. Next, by STRING database, protein-protein interaction (PPI) network was constructed. At the same time, hub genes in PPI network were identified. Then, some functional SNPs in hub genes that may affect gene expression have been annotated. Finally, we carried a study to explore the relationship between functional SNPs and NSCLC risk and overall survival in Chinese female non-smokers. A total of 488 DEGs were identified in our study. There are 29 proteins with a higher degree of connectivity in the PPI network, including *FOS*, *IL6* and *MMP9*. By using database annotation, we got 8 candidate functional SNPs that may affect the expression level of hub proteins. In the case-control study, we found that rs4754-T allele, rs959173-C allele and rs2239144-G allele were the protective allele of NSCLC risk. In dominant model, rs4754-CT+TT genotype were associated with a shorter survival time. In general, our study provides a novel research direction in the field of multi-omic data integration, and helps us find some critical genetic changes in disease.

## INTRODUCTION

Lung cancer is one of the most common malignant tumors and has a relatively poor 5-year relative survival rate in the world [1, 2]. There are two major forms of lung cancer: non-small cell lung cancer (NSCLC) and small cell lung cancer (SCLC), and NSCLC accounts for more than 80% of lung cancer. The exact mechanisms of underlying lung cancer are not fully elucidated. Smoking is considered to be a major environmental risk factor for lung cancer, but there are still 15% of male lung cancer cases and 53% of female lung cancer cases are not due to smoking [3]. A growing number of studies have indicated that genetic aberrations may be important in the genesis and development of human cancer [4–6]. Therefore, deep exploration of the relationship between genetic aberrations and NSCLC is needed to enhance risk prediction and improve prognosis.

The genesis and development of cancer is a multistage process which involves many genes and their interactions, and traditional studies that focus on single gene could no longer meet the demand any more. Microarray technology has been widely applied to global assessment of differentially expressed genes (DEGs) in many diseases. And then, by using bioinformatics method and experimental technology, the key genes involved in the pathogenesis of disease were found from candidate DEGs [7–9].

Any changes in DNA may influence the amino acid sequence or protein abundance. Single nucleotide polymorphisms (SNPs) are the most common type of genetic variation in human. It is characterized by a single nucleotide change in genome. The SNPs on exon usually brings the changes of amino acid sequence and further affect the function of protein. Those SNPs located at introns especially around 3′ untranslated regions (3′UTR), promoter elements and splicing sites are thought that they were likely to influence the expression level of proteins [10].

In this study, we analyzed the microarray data downloaded from Gene Expression Omnibus (GEO) and screened the DEGs between NSCLC and adjacent tissues. We then integrated DEGs results to carry out protein-protein interaction (PPI) network construction. Thereafter, we scanned those SNPs in the significant nodes (hub proteins) in PPI network, and found those functional SNPs may affect hub proteins level. At last, we systematically analyzed the association between these SNPs and NSCLC risk and overall survival.

## RESULTS

### DEGs analysis

**Figure 1:**
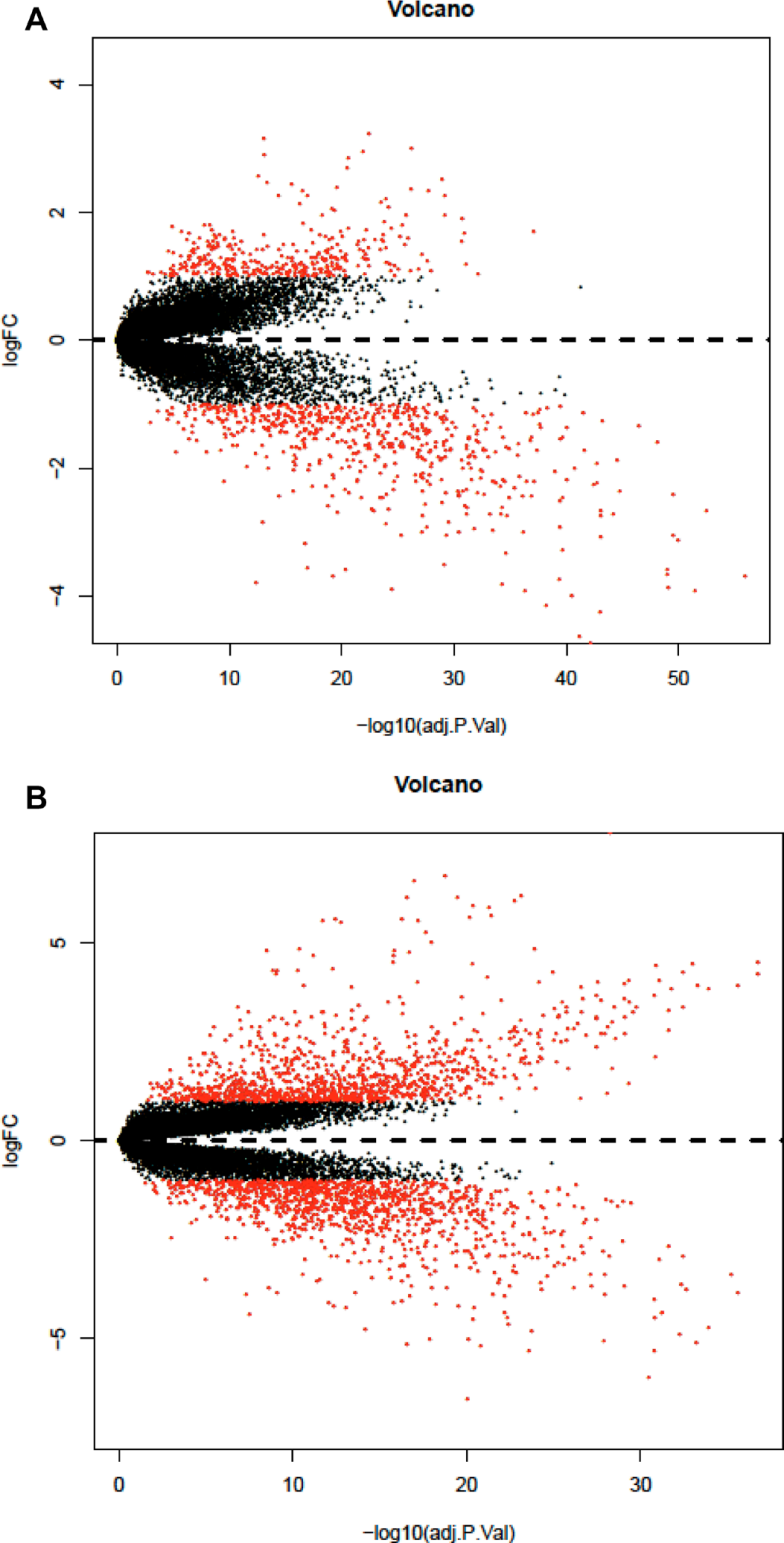
Volcano plot of differentially expressed genes (**A**) DEGs of lung adenocarcinoma (**B**) DEGs of lung squamous cell carcinoma.

Finally, we got 2295 DEGs in lung squamous cell carcinoma and 967 DEGs in lung adenocarcinoma, after the two groups of DEGs took the intersection, finally we got 488 DEGs (118 up-regulated and 370 down-regulated). Volcano plots for DEGs in lung adenocarcinoma and lung squamous cell carcinoma were shown in Figure 1.

### PPI network construction and hub genes in the PPI network

**Figure 2:**
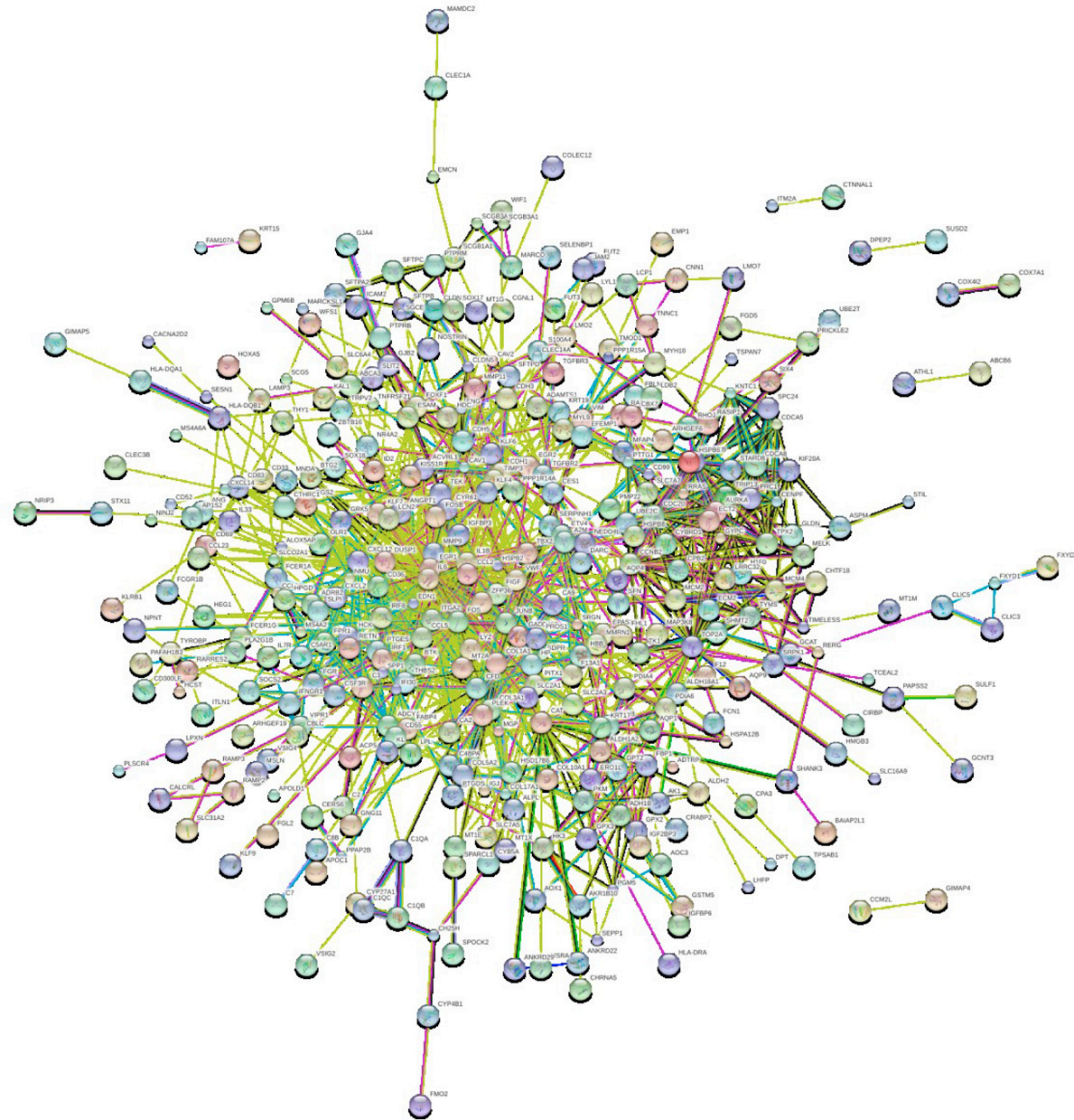
PPI network of differentially expressed genes (DEGs) Each node represents one DEG; edges indicate the interaction relationship.

**Figure 3:**
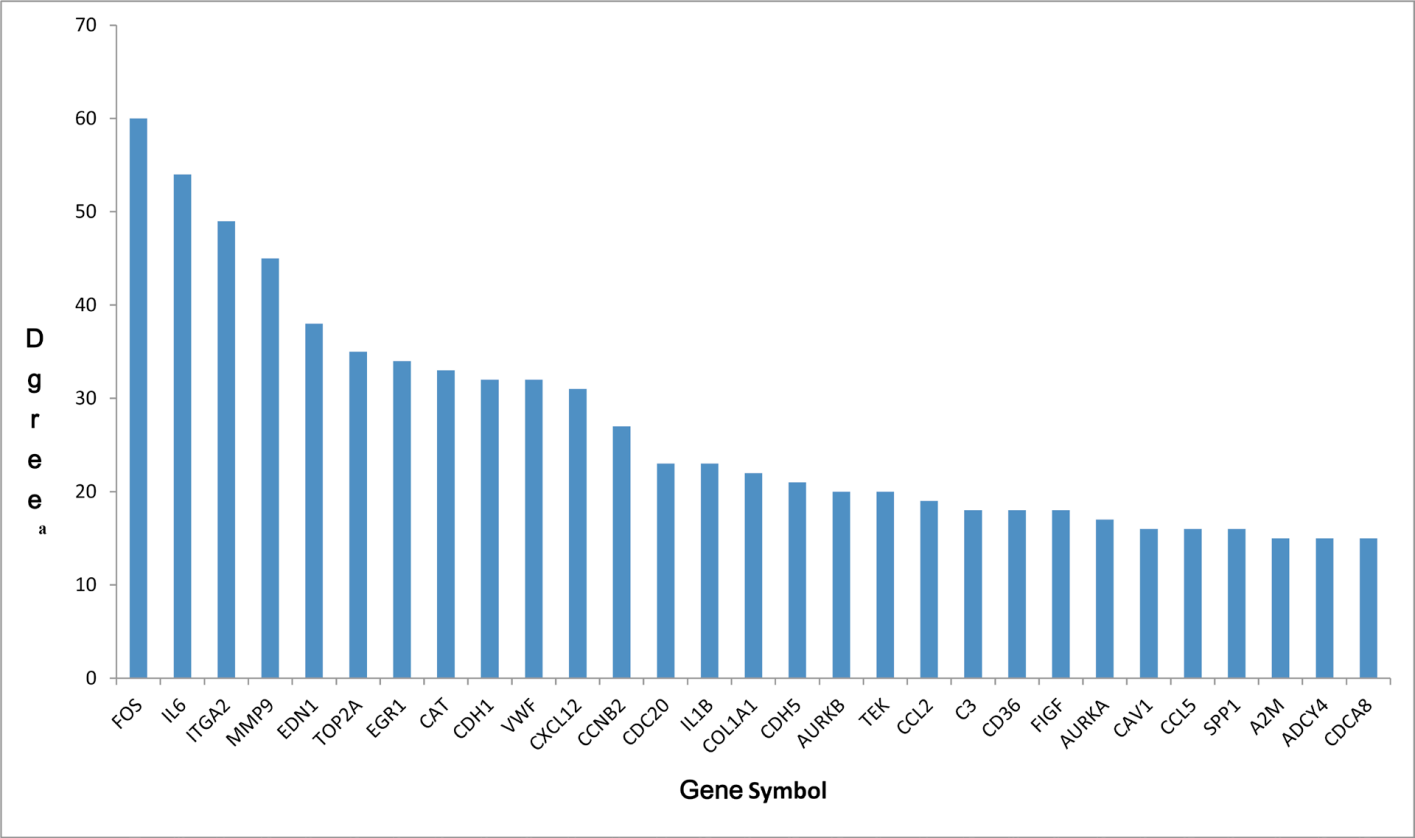
The hub genes in PPI network and their corresponding degree (**A**) The number of direct interactions of genes in the PPI network.

In order to further insight about the interaction between DEGs, we used STRING database to construct the PPI network. The PPI network (Figure 2) consisted of 376 nodes interacting by 2418 edges, the remaining 112 DEGs failed to form the PPI pairs. A great number of proteins interacting with others have relatively high degrees, which were considered as hub proteins, which are more likely to play a critical role in the genesis and development of cancer. The hub proteins and the number of their interactions were shown in Figure 3. There are 29 proteins whose degree is greater than 15, FOS (degree = 60) is the protein with the highest degree in the PPI network.

### Population characteristics

Finally 402 NSCLC patients and 395 cancer-free controls were included in the present study, the basic information of all subjects have been described in Table 1. All subjects were Chinese female non-smokers, and there was no significant difference in age between two groups (*p* = 0.692). Among cases, there were 322 adenocarcinomas, 66 Squamous cell carcinomas and 14 other tumors with a variety of different pathologies.

**Table 1:** Characteristics of NSCLC cases and cancer-free controls

Variables	Cases (%)	Controls (%)	*P* value
Females	402	395	
Mean age (years)	56.45 ± 11.45	56.13 ± 11.64	0.692
Histological			
Adenocarcinoma	322 (80.1%)		
Squamous cell carcinoma	66 (16.4%)		
Others^a^	14 (3.5%)		

^a^ including adenosquamous carcinoma, and large cell lung cancer.

### Results of SNPs selection

After database annotation, we selected 8 SNPs in hub genes which may be related to gene expression. The detail of the 8 SNPs is listed in Table 2. Among them, 1 SNPs located in 3′UTR region may fall into miRNA binding site; 2 SNPs located in splicing site; 2 SNPs may be an eQTL; and 3 SNPs were predicted fall intoTFBS.

**Table 2:** Single nucleotide polymorphism in hub genes

SNP	*Chr* location	Gene	position	Major/minor allele	Function predication
rs4754	chr4:88902691	SPP1	synonymous	C/T	Splicing (ESE or ESS)^a^
rs959173	chr7:116182053	CAV1	intron	T/C	eQTL^b^ + TFBS^b^
rs2069837	chr7:22768026	IL6	intron	A/G	TFBS^abc^
rs2066992	chr7:22768248	IL6	intron	T/G	TFBS^abc^
rs2239144	chr12:6196182	VWF	intron	G/T	TFBS^bc^
rs7306706	chr12:6215633	VWF	intron	G/A	eQTL^b^
rs3181385	chr14:24787587	ADCY4	3′UTR	T/C	miRNA binding site^a^
rs423490	chr19:6697405	C3	synonymous	G/A	Splicing (ESE or ESS)^a^

Abbreviations: ESE, exonic splicing enhancer; ESS, exonic splicing silencer; eQTL, expression Quantitative Trait Loci; TFBS, transcription factor binding site.

^a^predict by SNPinfo web server; ^b^ predict by Regulome DB database, ^c^ predict by HaploReg database.

### Genetic polymorphisms and NSCLC risk

Genotype distributions of the 8 SNPs are consistent with HWE in control group (*p* > 0.05). The distribution of genotypes and allele frequencies between cases and controls were summarized in Table 3. For rs4754, the A allele is a protective allele for NSCLC risk (adjusted OR = 0.762, 95% CI = 0.614–0.946, *p* = 0.014). Take rs4754-CC genotype as reference, TT genotype showed a relatively low risk of NSCLC (adjusted OR = 0.530, 95% CI = 0.317–0.884, *p* = 0.015). Compared with homozygous carriers of rs959173-TT genotype, TC genotype and TC + CC dominant model showed a lower risk of NSCLC (adjusted OR = 0.567, 95% CI = 0.347–0.928, *p* = 0.024; adjusted OR = 0.576, 95% CI = 0.354–0.936, *p* = 0.026, respectively). For rs2239144 we observed significant differences, the GT and TT genotypes were associated with a 1.508-fold (95%CI=1.105–2.058, *p* = 0.010) and 2.183-fold (95% CI = 1.450–3.287, *p* < 0.001) increased risk of NSCLC compared with GG genotype, T allele is a risk allele for NSCLC (adjusted OR = 1.513, 95% CI = 1.237–1.850, *p* < 0.001).

**Table 3:** Distribution of genotypes and ORs for NSCLC cases and cancer free controls

SNP	Genotype	NSCLC cases (%) *N* = 402	Controls (%) *N* = 395	*p* of HWE	Adjusted OR^a^	95% CI	*P*
Rs4754	CC	214 (53.2)	183 (46.3)	0.464	Ref		
	CT	160 (39.8)	167 (42.3)		0.820	0.612, 1.100	0.185
	TT	28 (7.0)	45 (11.4)		0.530	0.317, 0.884	0.015*
Dominant model					0.759	0.574, 1.002	0.052
Recessive model					0.583	0.356, 0.955	0.032*
Additive model	T allele				0.762	0.614, 0.946	0.014*
Rs959173	TT	373 (92.8)	348 (88.1)	0.686	Ref		
	TC	28 (7.0)	46 (11.6)		0.567	0.347, 0.928	0.024*
	CC	1 (0.2)	1 (0.3)		0.949	0.059, 15.327	0.971
Dominant model					0.576	0.354, 0.936	0.026*
Recessive model					1.019	0.063, 16.444	0.990
Additive model	C allele				0.600	0.376, 0.957	0.032*
Rs2069837	AA	260 (64.7)	264 (66.8)	0.548	Ref		
	AG	123 (30.6)	120 (30.4)		1.039	0.766, 1.408	0.806
	GG	19 (4.7)	11 (2.8)		1.754	0.819, 3.759	0.148
Dominant model					1.099	0.820, 1.473	0.527
Recessive model					1.731	0.813, 3.688	0.155
Additive model	G allele				1.141	0.888, 1.467	0.301
Rs2066992	TT	185 (46.0)	201 (50.9)	0.658	Ref		
	TG	174 (43.3)	159 (40.3)		1.185	0.883, 1.590	0.257
	GG	43 (10.7)	35 (8.9)		1.342	0.823, 2.190	0.239
Dominant model					1.213	0.918, 1.602	0.174
Recessive model					1.229	0.768, 1.965	0.390
Additive model	G allele				1.169	0.944, 1.447	0.152
Rs2239144	GG	124 (30.8)	169 (42.8)	0.270	Ref		
	GT	190 (47.3)	171 (43.3)		1.508	1.105, 2.058	0.010*
	TT	88 (21.9)	55 (13.9)		2.183	1.450, 3.287	< 0.001*
Dominant model					1.675	1.252, 2.240	0.001*
Recessive model					1.733	1.197, 2.509	0.004*
Additive model	T allele				1.513	1.237, 1.850	< 0.001*
Rs7306706	GG	168 (41.8)	154 (39.0)	0.064	Ref		
	GA	181 (45.0)	171 (43.3)		0.970	0.718, 1.313	0.845
	AA	53 (13.2)	70 (17.7)		0.695	0.457, 1.056	0.086
Dominant model					0.890	0.670, 1.181	0.419
Recessive model					0.705	0.479, 1.039	0.077
Additive model	A allele				0.855	0.698, 1.047	0.130
Rs3181385	TT	343 (85.3)	355 (89.9)	0.074	Ref		
	TC+CC	59 (14.7)	40 (10.1)		1.523	0.992, 2.337	0.054
Additive model	A allele				1.373	0.915, 2.061	0.126
Rs423490	GG	347 (86.3)	323 (81.8)	0.155	Ref		
	GA	54 (13.4)	71 (18.0)		0.708	0.482, 1.041	0.079
	AA	1 (0.2)	1 (0.3)		0.941	0.059, 15.126	0.966
Dominant model					0.711	0.485, 1.043	0.081
Recessive model					0.993	0.062, 15.939	0.996
Additive model	A allele				0.736	0.512, 1.058	0.098

Then, we performed a stratification analysis by pathological type. As shown in Supplementary Table 1, there were statistical differences between rs2239144, rs3181385, rs4754 and risk of lung adenocarcinoma. As the small sample size of squamous cell carcinoma in the present study, the significant associations on squamous cell carcinoma need to be validated in a large sample size population.

### Genetic polymorphisms and overall survival

**Figure 4:**
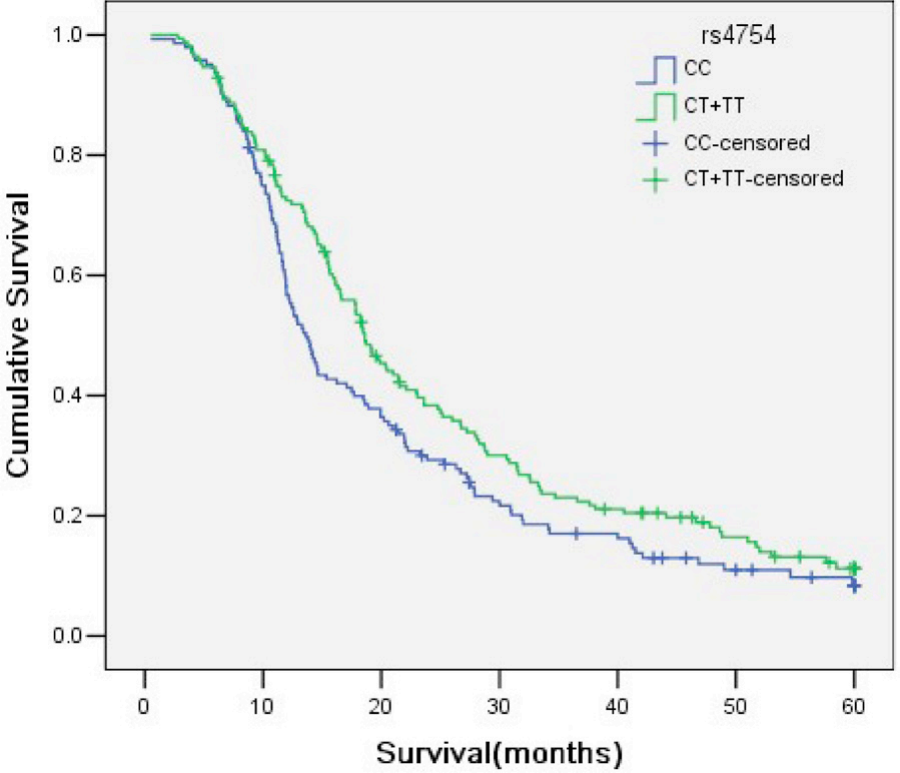
Genotypes of rs4754 SNP site in SPP1 and its association with NSCLC survival time

Of the patients in this study, there were 312 NSCLC patients with prognostic information. The results of the relationship between 8 SNPs and survival time were summarized in Table 4. Patients with rs4754-CC genotype showed a significantly longer survival time compared with those with CT or TT genotypes (25.124 months *vs*. 21.181 months), as shown in Figure 4. The other 7 SNPs didn't show any statistically significant correlation with survival time.

**Table 4:** Distribution of genotypes and survival time of patients

SNP	Genotype	NSCLC (%) (*n* = 312)	MST (mon)	Log-rank *P*	Adjusted HR^a^	95% CI
Rs4754	CC	168 (53.8)	25.124		Ref	
	CT	121 (38.8)	20.583	0.054	1.354	1.051,1.743*
	TT	23 (7.4)	24.172		1.037	0.638,1.685
Dominant model			21.181	0.039*	1.289	1.013,1.642*
Recessive model			23.218	0.625	0.908	0.567,1.454
Rs959173	TT	289 (92.6)	22.875		Ref	
	TC+CC	23 (7.4)	28.555	0.195	0.720	0.445,1.163
Rs2069837	AA	203 (65.1)	23.116		Ref	
	AG	94 (30.1)	22.876	0.552	1.013	0.777,1.319
	GG	15 (4.8)	28.470		0.717	0.379,1.357
Dominant model			23.627	0.811	0.968	0.751,1.248
Recessive model			23.039	0.278	0.711	0.378,1.338
Rs2066992	TT	142 (45.5)	23.086		Ref	
	TG	135 (43.3)	23.150	0.929	0.995	0.770,1.285
	GG	35 (11.2)	24.772		0.919	0.616,1.372
Dominant model			23.468	0.886	0.977	0.767,1.244
Recessive model			23.110	0.701	0.930	0.636,1.360
Rs2239144	GG	97 (31.1)	21.946		Ref	
	GT	138 (44.2)	23.096	0.262	0.923	0.698,1.220
	TT	77 (24.7)	25.583		0.770	0.556,1.068
Dominant model			23.972	0.255	0.860	0.666,1.110
Recessive model			22.517	0.125	0.808	0.606,1.075
Rs7306706	GG	134 (42.9)	23.807		Ref	
	GA	137 (43.9)	22.759	0.855	1.090	0.841,1.413
	AA	41 (13.1)	23.553		1.052	0.719,1.539
Dominant model			22.926	0.592	1.074	0.842,1.371
Recessive model			23.248	0.976	0.998	0.699,1.426
Rs3181385	TT	267 (85.6)	23.298		Ref	
	TC+CC	45 (14.4)	23.372	0.903	0.982	0.691,1.396
Rs423490	GG	268 (85.9)	23.821		Ref	
	GA+AA	44 (14.1)	19.818	0.197	1.250	0.889,1.758

## DISCUSSION

NSCLC is an aggressive and genomically unstable malignancy. A comprehensive genome-wide gene analysis by using bioinformatics and experimental methods to identify some potentially important genomic alterations is imperative. To begin with, we conducted a systematic study, which identified 488 overlapped DEGs from two microarray datasets (Lung squamous carcinoma and lung adenocarcinoma). Next, some hub proteins with a relatively high degree were confirmed in PPI network, and some SNPs that may affect the expression of hub proteins were identified by SNP annotation databases. Finally, we investigated these SNPs as potential contributor to genetic risk and survival of NSCLC.

The results in our study suggested that there were 29 proteins with a higher degree of connectivity in the PPI network, including FOS, IL6 and MMP9. In our study we found that *FOS* and *IL6* both with down regulation expression and they were the most significant hub proteins with degree of 60 and 54, respectively. In the previous study on FOS and lung cancer, some of the results were contradictory. One study on NSCLC found that c-FOS (a major member of the FOS family) was down regulation expression in malignant tissues compared with normal tissues. Another study found that the patients with higher expression level of c-FOS were corresponding with a shorter survival time [11]. More study should focus on the relationship between FOS and lung cancer to explore the mechanism between FOS and lung cancer. FOS family dimerize with JUN proteins to form AP-1 transcription factor complex, AP-1 could binding to the promoter and enhancer regions of target genes and regulate the transcription of target genes [11]. Previous study found that FOS overexpression can strongly enhance IL-6 to induced STAT3 transactivation, and involved in some cellular processes, including differentiation, proliferation and apoptosis [12]. Matrix metalloproteinases (MMPs) have been confirmed to be involved in the degradation of extracellular matrix components, which affect the physiological remodelling processes [13]. Our results show that MMP9 is relatively high expressed in lung cancer tissues. Previous research found that MMP9 was involved in lung-specific metastasis and was inducted by VEGFR-1 [14]. In lung carcinoma cell line, inactivation of MMP9 can inhibit tumor invasion [15]. Suggest us high expression of MMP9 may be associated with a poor prognosis in lung cancer

DNA genotype can affect gene expression, and gene expression can influence the onset and progression of diseases [16, 17]. Gene expression can be considered as a bridge between genotype and disease. In the human genome, SNP is the most universal genetic variant, which is a single base change at a specific site with the least allele frequency of 1% or greater [18]. SNPs in different gene regions will play different roles in biological processes, such as those non-synonymous SNPs in coding exons, which are considered to change the structure of protein by altering the amino acid sequence and further influence on diseases [19].

Alternative splicing of pre-mRNA is a critical regulatory mechanism for gene expression. Previous studies suggested that approximately 76% of genes produce alternatively spliced products, and about half of the transcript variants are caused by splicing variants [10, 20]. Abnormal splicing can affect mRNA and further influence the protein function. Some SNPs in exonic splice enhancer (ESE) or exonic splice silencer (ESS) have been confirmed to be likely to affect the risk of disease by causing aberrant splicing [21–24]. Secreted phosphoprotein 1 (SPP1) is a kind of important cytokine, which has been proved to play an important role in tumor progression and metastasis by regulating the cell signaling [25]. Rs4754 located at the fifth exon of SPP1 gene, and it was predicted located at ESE or ESS binding sites. Our study found that rs4754 could change the risk and survival of NSCLC. Previously, there were three studies on the relationship between rs4754 and cancer risk. The results of one study on gastric cancer are consistent with our findings that rs4754-C allele is a risk allele for cancer risk [26]. The results of the other two studies on nasopharyngeal carcinoma from a same Chinese population did not reach statistical significance [27, 28].

Transcription factor (TF) is a group of protein which can regulate gene expression and can be regarded as master regulators of gene expression. There are several factors that can affect the function of TF, such as availability of transcription factor binding site (TFBS) [29]. Some SNPs lie within the TFBS have been proved to be able to regulate gene expression by modif TFBS, such as abrogating an existing TFBS, creating a new TFBS or affecting the affinity between TF and TFBS [30–32]. IL-6 was initially thought to play a major role in immune and inflammatory responses, however IL6 abnormalities were found in many types of cancer, and some evidence showed that in cancer IL6 may play its downstream effects through JAK/STAT pathway [33–35]. Rs2069837 were predicted located at TFBS of IL6 gene. There are three articles about the association between rs2069837 and cancer risk, and their results consistently showed that the rs2069837-AA genotype was a protective factor for cervical cancer and hepatocellular carcinoma, one study found that rs2069837 were related to the IL6 expression level in cervical tissues. [36–38]. In our study the results were not statistically significant, further studies with lager sample size are needed to be conducted to explore the inconsistent result.

MiRNAs are short single-stranded noncoding RNAs, which regulate gene expression by post-transcriptionally regulation. MiRNAs through base pairing to the 3′UTR of target mRNAs lead to RNAs silencing [39]. SNPs located at miRNA binding sites can effect the base pairing between miRNA and target mRNA, which further affect miRNA-mediated genes expression. A number of studies have proved that SNPs mapping to miRNA binding sites can affect the expression level of target genes, thus involved in initiation and progression of disease [40–43]. Rs3181385 is a SNP located at miRNA binding site of *ADCY4* gene, in the present study there is a bordering significant association with the risk of NSCLC. There was no previous studies that have explored the relationship between rs3181385 and disease. Further studies are needed to verify the result.

eQTL is those SNPs that can regulate gene expression levels, and can be simply defined as the SNPs which were statistically associated with mRNA expression levels [44–46]. In the field of disease risk prediction and precision medicine, eQTL is likely to become a potentially high efficiency and effective biomarker. In our study, *CAV1* rs959173 was annotated as eQTL. One previously study found that rs959173-C allele was a protective allele and with a higher CAV1 protein level in systemic sclerosis patients. In our study, rs959173-C allele was a protective allele for NSCLC risk and the expression of CAV1 was down regulated in lung cancer tissue, which suggested us that rs959173 is likely to participate in the onset and development of NSCLC by affecting the expression of CAV1.

Over the last decade, genomewide association studies (GWAS) have identified a large number of disease-related SNPs covering more than 150 distinct diseases with a quite robust *p* value (*p* < 5 × 10^−8^). These disease-related SNPs, most of which we don't know how they affect the disease [44, 47]. Here we conducted a joint analysis to find out those SNPs which may affect diseases mediated by gene expression, and further explore the relationship between functional SNP and NSCLC risk and prognosis. Today, a very large amount of multi-omic data was produced along with the rapid development of biological technology. Life science has entered the post-genomic era, and how to effeciently process and integrate these biological information has become the problem that we should pay attention to. In general, our study provides a novel research direction in the field of multi-omic data integration.

## MATERIALS AND METHODS

### Data preprocessing and identification of DEGs

We systematically searched the GEO database (http://www.ncbi.nlm.nih.gov/geo/) with the following keywords and their combinations: “lung cancer, homo sapiens, expression profiling by array”. Finally, we selected two datasets suitable for our study. We downloaded the gene expression profiles of GSE18842 [48] and GSE32863 [49] from GEO. We included all the 32 lung squamous cell carcinoma samples and 32 adjacent non-tumor lung samples from the GSE18842 dataset. The GSE32863 dataset, we included 58 lung adenocarcinoma and 58 adjacent non-tumor lung tissues.

We downloaded the raw data from the GEO database. Logarithmic transformation (base 2) was performed on the expression value for a global normalization. When multiple probes corresponding to the same gene, average values of these probes were treated as the expression level of the gene. One probe corresponding to more than one gene, this value will be ignored as the nonspecificity.

The limma package [50] in R language was adopted to identify the DEGs between cancer samples and normal sample. Only genes exhibiting with adjusted *p* < 0.05 and | log_2_fold change (FC) | > 1.0 were selected as significant DEGs.

### PPI network construction

In order to reveal functional associations between proteins in a genome-wide scale, STRING online tool [51, 52] was used to construct a PPI network. In the PPI network, each node represents a protein, and each edge represents an interaction of pairwise proteins. The nodes with a relatively large number of edges were defined as hub proteins. In our study, the proteins with more than 15 edges were defined as hub protein.

### Study subjects and follow-up

In the present study, we recruited 402 NSCLC patients and 395 age matched (± 5 years) controls during March 2010 to May 2013 in accordance with the China Medical University Review Board approval. In order to control the impact of smoking, all participants included in our study were Chinese female non-smokers. All of them have signed the informed consent. Patients were recruited from the First Affiliated Hospital of China Medical University and Liaoning Cancer hospital, and controls were recruited from medical examination centers in the same hospital during the same period.

The clinical data was obtained from clinical records. Demographics and environmental exposure information were collected by face-to-face interviews. Each subject was drawn blood of 10 ml. Patients were followed up by telephone every 3 months until April 1st, 2015 to ensure that each patient has sufficient follow-up time. In the present study, death from NSCLC cancer is defined as the outcome event.

### SNPs selection and genotyping

Genomic DNA was isolated from blood samples by standard Phenol-chloroform Method. SNPs were genotyped by using the Illumina 660W SNP microarray (Illumina Inc San Diego, CA).

From dbSNP database, we obtained the candidate SNPs of those hub genes. Functional annotation of candidate SNPs were performed by SNPinfo web server [53], HaploReg resource V4.1 [54] and Regulome DB database [55]. We selected some SNPs that may affect gene expression with the following criterions: a. can be capture by Illumina 660 W SNP microarray probes; b. located at transcription factor binding site (TFBS), splicing sites or microRNA (miRNA) binding site; c. probably an expression Quantitative Trait Loci (eQTL); d. the minor allele frequency (MAF) > 0.05 in Chinese Han Beijing (CHB) population. and Followed these standards we finally got 8 SNPs which were investigated in the present study.

### Statistical analysis

Hardy-Weinberg's equilibrium (HWE) in controls was assessed by Pearson chi-squared test. Differences between cases and controls were calculated by *t*-test (continuous variable) or chi-squared text (categorical variable). The odds ratios (ORs) and their 95% confidence intervals (CIs) were calculated by logistic regression while adjusting for age to assess the relationship between SNP and lung cancer risk. Kaplan-Meier method and log-rank text were performed to evaluate the correlations between overall survival (OS) and genotypes. Hazard ratios (HRs) and their 95% CIs for OS were estimated by Cox proportionally hazards model. All data were analyzed by SPSS 22.0 (IBM, New York, NY, USA). A *p* < 0.05 was considered statistically significant.

## SUPPLEMENTARY MATERIALS TABLE


