# Supplementary material for: Multiple functional SNPs in differentially expressed genes modify risk and survival of non-small cell lung cancer in chinese female non-smokers
Source: Oncotarget. 2017 Jan 27;8(12):18924–34. doi: 10.18632/oncotarget.14836 (PMC5386658; doi:10.18632/oncotarget.14836)
Supplement: Supplementary file 1 [file oncotarget-08-18924-s001.pdf]

# Multiple functional SNPs in differentially expressed genes modify risk and survival of non-small cell lung cancer in chinese female non-smokers

## Supplementary Materials

### Supplementary Table 1: Distribution of genotypes and ORs for different types of NSCLC and controls

| SNP | Genotype | Controls (%) $N$ = 395 | Adenocarcinoma (%) $N$ = 322 | Adjusted OR[a] | 95% CI | $P$ | Squamous cell carcinoma $N$ = 66 | Adjusted OR[a] | 95% CI | $P$ |
|---|---|---|---|---|---|---|---|---|---|---|
| Rs4754 | CC | 183 (46.3) | 163 (50.6) | Ref | | | 42 (63.6) | Ref | | |
| | CT | 167 (42.3) | 136 (42.2) | 0.915 | 0.671,1.246 | 0.572 | 20 (30.3) | 0.526 | 0.297,0.933 | 0.028* |
| | TT | 45 (11.4) | 23 (7.1) | 0.566 | 0.328,0.978 | 0.042* | 4 (6.1) | 0.383 | 0.130,1.124 | 0.081 |
| Dominant model | | | | 0.842 | 0.627,1.130 | 0.252 | | 0.495 | 0.289,0.849 | 0.011* |
| Recessive model | | | | 0.597 | 0.353,1.011 | 0.055 | | 0.494 | 0.171,1.424 | 0.191 |
| Additive model | T allele | | | 0.817 | 0.651,1.025 | 0.080 | | 0.558 | 0.358,0.869 | 0.010* |
| Rs959173 | TT | 348 (88.1) | 295 (91.6) | Ref | | | 64 (97.0) | Ref | | |
| | TC+CC | 47 (11.9) | 27 (8.4) | 0.678 | 0.412,1.115 | 0.126 | 2 (3.0) | 0.238 | 0.056,1.003 | 0.051 |
| Additive model | C allele | | | 0.702 | 0.435,1.133 | 0.148 | | 0.239 | 0.057,0.996 | 0.049* |
| Rs2069837 | AA | 264 (66.8) | 211 (65.5) | Ref | | | 40 (60.6) | Ref | | |
| | AG | 120 (30.4) | 96 (29.8) | 1.001 | 0.724,1.384 | 0.994 | 22 (33.3) | 1.213 | 0.690,2.130 | 0.503 |
| | GG | 11 (2.8) | 15 (4.7) | 1.705 | 0.767,3.792 | 0.190 | 4 (6.1) | 2.356 | 0.714,7.774 | 0.159 |
| Dominant model | | | | 1.061 | 0.777,1.448 | 0.710 | | 1.311 | 0.767,2.243 | 0.322 |
| Recessive model | | | | 1.705 | 0.772,3.766 | 0.187 | | 2.220 | 0.684,7.201 | 0.184 |
| Additive model | G allele | | | 1.110 | 0.851,1.449 | 0.441 | | 1.341 | 0.859,2.095 | 0.197 |
| Rs2066992 | TT | 201 (50.9) | 150 (46.6) | Ref | | | 28 (42.4) | Ref | | |
| | TG | 159 (40.3) | 137 (42.5) | 1.154 | 0.845,1.576 | 0.369 | 30 (45.5) | 1.357 | 0.778,2.365 | 0.282 |
| | GG | 35 (8.9) | 35 (10.9) | 1.361 | 0.813,2.279 | 0.241 | 8 (12.1) | 1.571 | 0.660,3.742 | 0.307 |
| Dominant model | | | | 1.190 | 0.886,1.599 | 0.247 | | 1.403 | 0.828,2.376 | 0.208 |
| Recessive model | | | | 1.256 | 0.767,2.058 | 0.365 | | 1.389 | 0.612,3.150 | 0.432 |
| Additive model | G allele | | | 1.162 | 0.972,1.457 | 0.193 | | 1.303 | 0.882,1.924 | 0.184 |
| Rs2239144 | GG | 169 (42.8) | 105 (32.6) | Ref | | | 18 (27.3) | Ref | | |
| | GT | 171 (43.3) | 147 (45.7) | 1.384 | 0.996,1.922 | 0.053 | 32 (48.5) | 1.734 | 0.933,3.223 | 0.082 |
| | TT | 55 (13.9) | 70 (21.7) | 2.063 | 1.342,3.171 | 0.001* | 16 (24.2) | 2.730 | 1.302,5.723 | 0.008* |
| Dominant model | | | | 1.548 | 1.139,2.103 | 0.005* | | 1.989 | 1.116,3.543 | 0.020* |
| Recessive model | | | | 1.721 | 1.166,2.540 | 0.006* | | 1.992 | 1.059,3.747 | 0.033* |
| Additive model | T allele | | | 1.458 | 1.178,1.804 | 0.001* | | 1.705 | 1.176,2.472 | 0.005* |
| Rs7306706 | GG | 154 (39.0) | 137 (42.5) | Ref | | | 27 (40.9) | Ref | | |
| | GA | 171 (43.3) | 139 (43.2) | 0.920 | 0.667,1.268 | 0.610 | 33 (50.0) | 1.109 | 0.637,1.929 | 0.715 |
| | AA | 70 (17.7) | 46 (14.3) | 0.727 | 0.470,1.125 | 0.152 | 6 (9.1) | 0.484 | 0.191,1.226 | 0.126 |
| Dominant model | | | | 0.862 | 0.639,1.163 | 0.332 | | 0.922 | 0.542,1.568 | 0.764 |
| Recessive model | | | | 0.761 | 0.508,1.139 | 0.185 | | 0.451 | 0.187,1.086 | 0.076 |
| Additive model | A allele | | | 0.857 | 0.691,1.062 | 0.159 | | 0.789 | 0.536,1.163 | 0.232 |
| Rs3181385 | TT | 355 (89.9) | 272 (84.5) | Ref | | | 60 (90.9) | Ref | | |
| | TC + CC | 40 (10.1) | 50 (15.5) | 1.771 | 1.125,2.789 | 0.014* | 6 (9.1) | 0.945 | 0.382,2.339 | 0.902 |
| Additive model | A allele | | | 1.464 | 0.960,2.232 | 0.076 | | 0.823 | 0.343,1.974 | 0.662 |
| Rs423490 | GG | 323 (81.8) | 274 (85.1) | Ref | | | 59 (89.4) | Ref | | |
| | GA + AA | 72 (18.2) | 48 (14.9) | 0.786 | 0.527,1.171 | 0.237 | 7 (10.6) | 0.540 | 0.237,1.232 | 0.143 |
| Additive model | A allele | | | 0.809 | 0.554,1.180 | 0.271 | | 0.550 | 0.247,1.222 | 0.142 |
